# Repurposing Agents as Anti-Infective Therapeutics to Aid in the Treatment of *Candida auris* Infections

**DOI:** 10.3390/idr17060144

**Published:** 2025-11-27

**Authors:** Nazary Nebeluk, James B. Doub

**Affiliations:** 1 The Doub Laboratory of Translational Bacterial Research, University of Maryland School of Medicine, Baltimore, MD 21201, USA; 2 Division of Clinical Care and Research, Institute of Human Virology, University of Maryland School of Medicine, Baltimore, MD 21201, USA

**Keywords:** *Candida auris*, nosocomial infections, multidrug resistance, lavage solutions, N-acetylcysteine

## Abstract

Background: *Candida auris* is an emerging nosocomial fungal pathogen whose inherent multidrug resistance and ability to form biofilms make treatment extremely difficult. Given the limited number of therapeutic options available and the poor clinical outcomes associated with current therapeutics, this study evaluated the potential of repurposing existing agents to treat *C. auris* infections. Methods: Six clinical *C. auris* isolates from a single tertiary care center were tested for in vitro susceptibility to topical agents (hypochlorous acid, chlorhexidine gluconate, sodium hypochlorite) and systemic agents (N-acetylcysteine, ethylenediaminetetraacetic acid, ethyl pyruvate). Furthermore, these six isolates were allowed to form biofilms and the ability of repurposed agents to disrupt *C. auris* biofilms was measured. Results: All agents except N-acetylcysteine demonstrated inhibitory activity against planktonic *C. auris.* With respect to *C. auris* biofilms, these were characterized using electron microscopy and all six agents showed statistically significant (*p* < 0.05) ability to disrupt biofilms over controls. Moreover, the ability to disrupt biofilms was also statistically significant (*p* < 0.05) when compared to use of either normal saline or amphotericin B. Discussion: These findings support the potential clinical utility of repurposing existing agents, such as Ethyl Pyruvate or EDTA, for systemic *C. auris* infections, or hypochlorous acid for *C. auris* wound infections. Yet, further studies are needed to optimize dosing parameters and evaluate in vivo efficacy and tolerability.

## 1. Introduction

Nosocomial infections impact 4% of all hospitalized patients in the United States [1]. These are often difficult to treat given nosocomial pathogens often possess numerous virulence factors that can be associated with multidrug resistance [1]. Fungal nosocomial infections are particularly difficult to treat given the limited number of antifungal therapeutics currently available. Treatment of this syndrome is further complicated in cases of *Candida auris* infections, as this microbe can be resistant to many of the traditional antifungal therapies such as fluconazole and amphotericin [2]. This pathogen was first discovered in Japan in 2009 and has caused over 3000 infections in the United States of America with an increasing disease burden [3]. Therefore, novel therapeutics are needed to aid in the treatment to thereby reduce morbidity and mortality.

However, there is heterogeneity in nosocomial *C. auris* infections. Some infections are associated with only planktonic states while others occur with deep-seated biofilms on prosthetic material. This further complicates treatment because once *C. auris* forms biofilms on implanted hardware or native tissue, conventional antifungals have limited ability to disrupt those biofilms [4]. Thus, the aims of this study were to evaluate the potential to repurpose agents currently used in other clinical settings to aid in eradicating *C. auris* biofilm and planktonic states.

## 2. Materials and Methods

### 2.1. C. auris Clinical Isolates

This study was approved by the University of Maryland Internal Review Board (HP-00114224, approved on 2 April 2025) and Institutional Biosafety Committee (IBC-00007724, approved on 7 April 2025). Six *C. auris* clinical isolates that were preserved at −80 °C were used in this study. Three were from chronic wound infections, overlying the sternum, sacrum, and hip, respectively, and three were from deep-seated infections associated with fungemia and a vascular graft infection, endocarditis, and osteomyelitis.

### 2.2. Repurposed Clinical Agents

Not all repurposed agents have the potential to be administered intravenously given associated systemic toxicities. As a result, we divided potential repurposed agents into topical agents and systemic agents. Potential topical antimicrobial agents used in this study were devised based on data from use of these agents as irrigating solutions in orthopedic infections [5]. The selected topical agents included 1% hypochlorous acid (Vashe) (10,000 µg/mL), 0.04% (400 µg/mL) chlorohexidine gluconate (CHG), and 0.5% sodium hypochlorite (5000 µg/mL).

The intravenous agents evaluated were N-acetylcysteine (NAC), Ethylenediamine-tetraacetic acid (EDTA), and ethyl pyruvate. Clinically relevant concentrations of each of these were devised from prior clinical trials using these agents as adjuvants for other indications. For EDTA, this was determined from clinical trials that used this agent in chelation therapy to treat patients suffering from cardiac infarctions in which doses of 3 g in 500 mL of normal saline were used [6]. Ethyl pyruvate doses were devised from a murine study that used this agent to reduce inflammation in pancreatitis for which 40 mg/kg were used every 6 h; this dose was extrapolated to an 80 kg human for which we devised a dose of 2.5 mg/mL [7]. Finally, N-acetylcysteine doses were devised from the use of this agent in the treatment of acetaminophen-induced liver injury in which 10% concentrations are routinely used [8].

### 2.3. Determination of Anti-Planktonic Activity

To assess the activity of these agents against planktonic *C. auris* states, the six clinical isolates were grown overnight in Sabouraud dextrose broth (SAB) at 37 °C. The overnight growth was then serially diluted to 1 × 10^6^ CFU/mL and placed into a 48-microwell plate that had 500 µL of SAB present. A total of 1% Vashe, 0.04% CHG, 0.5% sodium hypochlorite, EDTA 2500 µg/mL, ethyl pyruvate 2500 µg/mL, and N-acetylcysteine 10% were then added to individual wells. These agents were then serially diluted to concentrations of 10^−5^ and each concentration was placed into wells of the microwell plates. Controls were used, a SAB only negative control and a SAB and *C. auris* alone positive control. Also, fluconazole, micafungin, and amphotericin B were used as comparisons and were serially diluted from 512 µg/mL to 0.25 µg/mL. To conduct this experiment, all plates were incubated for 16 h at 37 °C and then evaluated for growth inhibition. This was conducted by comparing the optical density (600 nm) of controls to clinical agents with a SpectraMax iD5 (Molecular devices, Sunnyvale, CA, USA). This experiment was conducted in duplicates and repeated for each of the six clinical isolates.

### 2.4. Visualization of C. auris Biofilm and Effect of Agents on Biofilm Dispersal

*C. auris* isolates were grown overnight in SAB at 37 °C. One hundred μL of overnight growth was then taken and allowed to grow to an optical density corresponding to 1 × 10^6^ CFU/mL. Then, 1 mL of fungal growth was added to 24 microwell plates (Falcon, Corning, NY, USA) with 1 mL of SAB. The microwell plates were then incubated at 37 °C for 48 h. This was repeated for all clinical isolates and conducted in duplicate. Also, *C. auris* grew in the same fashion in wells that contained 1 cm plastic cones and 1 cm steel solid cylinders to be used as surfaces for adherence to visualize biofilms with scanning electron microscopy (SEM).

After 48 h the liquid components were discarded by inverting the plates. The wells were then stained with crystal violet for 10 min. Plates were then inverted and washed with normal saline. Lavage solutions of each agent were then prepared with same concentrations used in planktonic assay along with comparisons including amphotericin B (4 µg/mL) and normal saline with no chemical agent added. Two mL of each lavage solution was added to their corresponding wells. The negative control did not undergo lavage to allow for maximum fungal growth. The solutions were left to dwell for 30 min. Plates were then inverted and washed with normal saline and then 30% acetic acid was added to the microwell plates. Optical density was then measured to assess biofilm disruption compared to saline and growth controls by use of a SpectraMax iD5 (Molecular devices, Sunnyvale, CA, USA). Statistical comparisons were calculated with the use of Graphpad Prism (Version 100.0 for Windows, Dotmatics, Boston, MA, USA). in which Chi-squared testing was utilized for comparison. A *p* value < 0.05 was considered significant.

SEM was used to visualize *C. auris* biofilms. After biofilms were grown as stated above, the plastic cones and steel cylinders were moved to new microwell plates and treated with SEM fixative (2% paraformaldehyde and 2.5% glutaraldehyde). These were left overnight and then dehydrated and sputter coated with platinum, followed by SEM visualization (FEI Quanta 200, Hillsboro, OR, USA) with assistance from the University of Maryland, School of Medicine Center for Innovative Biomedical Resources Electron Microscopy Core Imaging Facility.

## 3. Results

For the three standardized antifungal antimicrobials, amphotericin B showed the greatest intrasample variability ranging from 0.5 µg/mL to 64 µg/mL, with four of the six samples falling below the current breakpoint for susceptibility (2 µg/mL). All six of the clinical strains were resistant to fluconazole as all had MIC above the breakpoint of 32 µg/mL. This is different from the micafungin susceptibility, in which all six strains were susceptible to micafungin (as defined by resistance > 0.25 µg/mL). When evaluating the ability of the repurposed clinical agents to limit planktonic *C. auris* growth, we found that all six clinical isolates were susceptible to inhibition by Vashe, CHG, sodium hypochlorite, and ethyl pyruvate without intrasample variability (Table 1). For Vashe, this was observed at 0.1% (1000 µg/mL), or a tenth of the dose currently used in orthopedic infections, and ethyl pyruvate also demonstrated inhibition at its clinically studied dose of 2.5 mg/mL. For CHG and sodium hypochlorite, this concentration was a hundredth of the current clinical concentration (0.04% and 0.05%, respectively). EDTA also showed a variable response, with four samples showing inhibition at 250 µg/mL and two samples showing inhibition at a log higher dose of 2500 µg/mL. Notably, NAC did not show any significant growth inhibition of planktonic *C. auris*.

**Figure 1:**
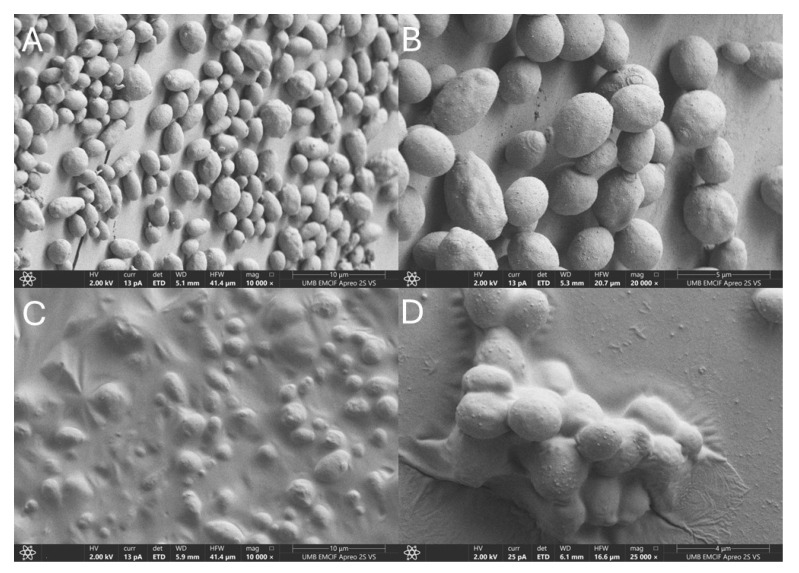
Representative high magnification scanning electron microscopy images of *C. auris* clinical isolates grown on plastic pipette tips: Images of 48 h *C. auris* biofilm growth on plastic pipette tips shows two distinct morphologies with some isolates growing as a sheet of independent yeast cells (**A**,**B**) and others excreting an extracellular polysaccharide matrix (**C**,**D**) that envelops clusters of yeast cells.

**Figure 2:**
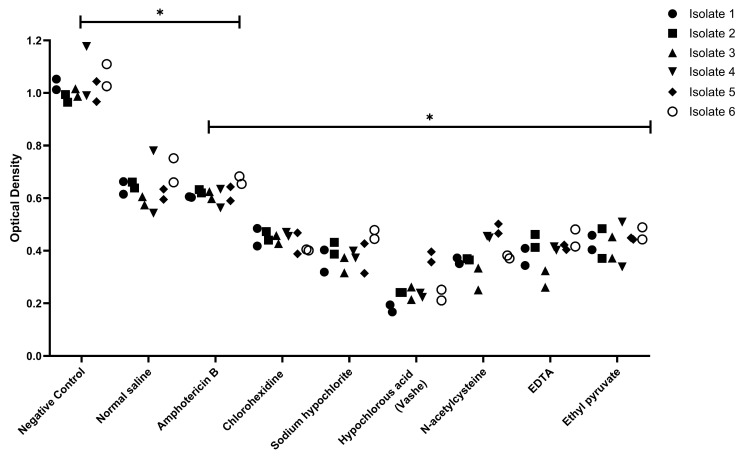
*C. auris* biofilm reduction as measured by optical density after exposure to repurposed clinical agents. Negative control plates did not receive any lavage. There was a significant reduction (*p* < 0.05) in optical density observed after administration of normal saline, amphotericin B. Further statistically significant reductions were seen when comparing administration of all six of the repurposed agents (*p* < 0.05) to normal saline or amphotericin B. * *p* < 0.05. EDTA: Ethylenediaminetetraacetic acid.

With respect to *C. auris* biofilms, we observed that there were two distinct morphotypes of *C. auris* biofilm formation with some clinical samples showing individual yeast biofilms with light aggregation (Figure 1A,B) and other samples expressing an extracellular polysaccharide matrix to produce a covering (Figure 1C,D). Despite this variation under electron microscopy, we found similar responses when evaluating the ability of clinical agents to disperse *C. auris* biofilms (Figure 2). Compared to the no intervention negative control, normal saline or amphotericin B significantly reduced optical density, reflecting a disruption of the biofilm which we expected given mechanical lavage itself causes biofilm disruption (*p* < 0.05). However, all six of the clinical agents led to a statistically significant increase in biofilm dispersion (*p* < 0.05), more than what was observed when these values of residual biofilm were compared with normal saline or amphotericin B lavage.

## 4. Discussion

*C. auris* has been an emerging pathogen of great clinical concern since it was first discovered in 2009. Often found in healthcare settings, its inherent multidrug resistance to azoles, polyenes, and rising echinocandin resistance make treatment difficult for both systemic and localized infections [2]. As a result, there is a critical need for new therapeutics to treat *C. auris* infections. Obstinately, the development cycle of new antifungal therapeutics is long and immensely costly to pharmaceutical companies, resulting in a limited pipeline of antifungal therapeutics currently being evaluated. Consequently, it may be more prudent to evaluate the ability of repurposing existing agents used in clinical medicine to aid in the treatment of *C. auris* infections.

In this study, we found that all the clinically studied agents had efficacy in limiting planktonic *C. auris* growth except for NAC. These findings are expected for potential topical agents (hypochlorous acid, chlorhexidine gluconate, and sodium hypochlorite) which are known disinfectants and are used in clinical practice already. Hypochlorous acid, tradename Vashe, mimics the oxidate burst used by neutrophils and causes disruption of the fungal membrane [9]. Chlorhexidine gluconate is a cationic bisbiguanide that binds to cell walls, destabilizing them and causing lysis [10]. Sodium hypochlorite, a diluted bleach with the eponym Dakin’s solution, acts as an oxidizing agent and causes disruption of the fungal cell wall [10]. In our study, hypochlorous acid was efficacious at a concentration of 0.1% (1000 µg/mL) or a tenth of the dose currently used in orthopedic infections [5], while CHG and sodium hypochlorite were efficacious at a concentration that was a hundredth of the current clinical concentration (0.04% and 0.05%, respectively). There was no difference in effective dose across all six clinical samples. The ability of such dilute solutions to have an inhibitory effect likely reflects their ability to make direct contact with the organism in this ex vivo model.

CHG, sodium hypochlorite, and Vashe have significant systemic toxicities if given intravenously and thus could not be used with systemic administration. Currently, amphotericin B is the antifungal agent recommended for severe systemic Candidal infections [11] and thus was used as the comparison in these experiments. However, it should be noted that echinocandins are the recommended agents for less disseminated *C. auris* infections given the limited resistance to these agents as seen here with all strains having MIC ≤ 2 µg/mL [12]. Here, amphotericin B demonstrated efficacy against four of the six clinical isolates, with isolates 2 and 5 showing minimum inhibitory concentrations (MIC) greater than the 2 µg/mL cutoff traditionally used to determine clinical susceptibility. This finding appears to be in line with what is seen in clinical practice, where up to 50% of isolates are resistant to amphotericin B [13].

As a result, we evaluated the potential of repurposing several agents that could be used systemically—EDTA, NAC, and ethyl pyruvate. Ethyl pyruvate showed consistent inhibition at 2.5 mg/mL, likely via disruption of the fungal glucose oxidation pathway, which could indicate no significant variation amongst clinical isolate carbon metabolism. Notably, our inhibitory concentration is consistent with prior studies that showed ethyl pyruvate can inhibit planktonic colonies of several different *Candidal* spp. isolated from vaginal flora at a MIC of 25 mM (~2900 µg/mL) [14]. EDTA had a variable response, with four samples showing inhibition at 250 µg/mL and two samples showing inhibition at a log higher dose of 2500 µg/mL. Notably, these two samples (isolate 2 and 5) also needed higher doses of amphotericin B for growth inhibition (32 µg/mL and 64 µg/mL, respectively). EDTA is a metal ion chelator and causes cell membrane disruption; notably, amphotericin B causes formation of pores on the fungal cell wall that allow rapid loss of intracellular ions, contributing to cell death. It is possible that the same porin or structural mutation plays a role in retaining cationic molecules and increasing resistance to both agents.

Interestingly, we did not find any antifungal effects of NAC against the planktonic states of our clinical isolates. This is despite prior work showing that nanoparticles containing NAC had an inhibitory effect on clinical *C. auris* isolates via generation of nitrous oxide (NO) [15]. We hypothesize this maybe occurred because NAC was not protected like in the prior study and was degraded rapidly prior to having a significant clinical effect. Likewise, the dispersion of NO throughout the medium might not have been sufficient to inhibit fungal growth. Nonetheless, further studies are needed to clarify these findings and hypotheses.

While anti-planktonic state activity is important, much of the virulence of *C. auris* infections is driven by biofilm formation. Using SEM, we observed that on foreign materials, *C. auris* formed two unique biofilm morphotypes. The first is represented in Figure 1 panels A/B and shows the individual yeast cells in high resolution; the second is represented in panels C/D and shows the yeast covered by a thin extracellular matrix, presumably of polysaccharide composition. This highlights the unique nature of *C. auris* biofilms as they do not show identifiable hyphae or pseudohyphae expression in their biofilm state, unlike other *Candidal* spp. such as *albicans*, *tropicalis*, and *dublinesis* [16,17]. Despite the differences seen visually using SEM, there was no significant difference in biofilm response to treatment with clinical agents. We found that simple lavage with either normal saline or amphotericin B led to a significant reduction in biofilm formation in all six clinical samples tested (Figure 2). This occurred because of the mechanical shearing force of the intermittent lavage of all solutions added. This has been observed previously in vitro in other disease states where we have found that repeat instillation cycles can contribute to decreased pathogen burden [18]. Of note, all six repurposed agents in our study led to statistically significant decreases in biofilm burden (*p* ≤ 0.05) compared to normal saline or amphotericin B. This likely reflects the direct cytotoxic effects of these chemicals coming in contact with the cell surface. Moreover, this suggests that these agents are able to outperform amphotericin B in biofilm dispersion as this agent relies on fungal replication for maximal therapeutic efficacy.

Based on these findings, we believe that treatment of *C. auris* wound infections could be aided with irrigation with a topical agent such as hypochlorous acid, CHG, or sodium hypochlorite. This would provide clinical benefit over irrigation with normal saline or amphotericin B. In trauma patients, previous reports have indicated the utility of irrigation via a vacuum-assisted closure device for prevention of invasive fungal disease progression [19]. While not statistically significant, our findings indicate that Vashe may have the most significant effect on local disease burden, consistent with prior literature showing its ability to disrupt biofilms in other *Candidal* spp. [2]. Given its affordability and minimal toxicity, there likely would be little harm in adopting this clinical practice, although we recognize further studies with animal or clinical models are necessary. As stated previously, Vashe cannot be used for systemic infections due to toxicity. Therefore, in deep-seated cases ethyl pyruvate or EDTA may provide a clinical benefit as an adjunct to antifungal therapy, but further investigation is needed.

While this study provides support for repurposing several existing clinical agents to aid in treating *C. auris* infections, there are a few limitations. For one, we only evaluated six clinical samples from a single tertiary care center. Given the rarity of this infection, follow-up collaborative multicenter studies would be prudent to ensure a wide array of clinical isolates have similar susceptibilities to these repurposed agents. This is particularly important as EDTA showed a variable inhibitory concentration within even our sample set and therefore certain agents may need MIC established for clinical use. Secondly, the concentrations of agents used were extrapolated from common clinical usages of these agents. Follow-up studies to determine optimal antifungal concentrations would be advantageous. Correlated with this is the fact that we only evaluated six potential agents, but additional agents, such as methylene blue, could have clinical potential. This study therefore should be used as a catalyst for others to consider repurposing other agents to aid in the treatment of difficult to treat infections. Lastly, we used amphotericin B as a comparison instead of echinocandins, given that this is the recommended agent to use for severe candidal infections, but further studies using micafungin as comparison should also be conducted.

## 5. Conclusions

In conclusion, *C. auris* is an emerging pathogen with limited antifungal therapeutic options. Here, we evaluated the ability to repurpose agents to aid in the treatment of *C. auris* infections. All agents evaluated were able to disrupt *C. auris* biofilm more than normal saline or amphotericin B lavage. Moreover, all agents except NAC had activity against planktonic *C. auris.* Overall, this study should spearhead follow-up research into repurposing additional agents for *C. auris* and other emerging hard to treat infections to reduce morbidity and mortality.

## Figures and Tables

**Table 1:** Minimal inhibitory concentrations of novel agents against planktonic *C. auris* clinical isolates.

	*C. auris* 1	*C. auris* 2	*C. auris* 3	*C. auris* 4	*C. auris* 5	*C. auris* 6
Fluconazole	512 µg/mL	512 µg/mL	256 µg/mL	256 µg/mL	512 µg/mL	512 µg/mL
Amphotericin B	1 µg/mL	32 µg/mL	0.5 µg/mL	0.5 µg/mL	64 µg/mL	1 µg/mL
Micafungin	0.25 µg/mL	0.25 µg/mL	0.25 µg/mL	0.125 µg/mL	0.125 µg/mL	0.25 µg/mL
Hypochlorous acid (Vashe)	1000 µg/mL	1000 µg/mL	1000 µg/mL	1000 µg/mL	1000 µg/mL	1000 µg/mL
Chlorhexidine	4 µg/mL	4 µg/mL	4 µg/mL	4 µg/mL	4 µg/mL	4 µg/mL
Sodium hypochlorite	50 µg/mL	50 µg/mL	50 µg/mL	50 µg/mL	50 µg/mL	50 µg/mL
N-acetylcysteine ^2^	>100,000 µg/mL	>100,000 µg/mL	>100,000 µg/mL	>100,000 µg/mL	>100,000 µg/mL	>100,000 µg/mL
Ethyl pyruvate	2500 µg/mL	2500 µg/mL	2500 µg/mL	2500 µg/mL	2500 µg/mL	2500 µg/mL
EDTA ^1^	250 µg/mL	2500 µg/mL	250 µg/mL	250 µg/mL	2500 µg/mL	250 µg/mL

^1^ EDTA: Ethylenediaminetetraacetic acid; ^2^ no growth inhibition at doses evaluated of N-acetylcysteine.

## Data Availability

The data generated and analyzed during the current study are available upon reasonable request from the corresponding author.
